# Reply to comment on “Association between self‐reported weight loss and new long‐term care insurance certifications: A 9‐year Japanese older adult cohort study”

**DOI:** 10.1111/ggi.70070

**Published:** 2025-05-09

**Authors:** Ryo Katayose, Mika Okura, Hidenori Arai, Mihoko Ogita

**Affiliations:** ^1^ Department of Clinical Nursing Shiga University of Medical Science Otsu Japan; ^2^ Department of Nursing Kyoto College of Nursing Kyoto Japan; ^3^ Department of Nursing Yamanashi Prefectural University Kofu Japan; ^4^ National Center for Geriatrics and Gerontology Obu Japan

Dear Editor,

We sincerely appreciate Yilmaz Kars *et al*.'s interest in and comments^1^ on our study.^2^ They raised four concerns regarding our study: measurement bias in self‐reported surveys among individuals with dementia or cognitive impairment; the need to examine comorbidities and medication use separately; the handling of malignancies as a critical confounder; and the background of weight loss as well as the obesity paradox. We address these concerns as follows.

Regarding the first concern – measurement bias in self‐reported surveys among individuals with dementia or cognitive impairment – we acknowledge that this bias may exist in our study. It is possible that individuals with dementia or cognitive impairment exhibited lower response accuracy not only for the primary variable (weight loss) but also for all other variables. However, a notable strength of our study is that participants were not certified for long‐term care insurance (LTCI), had no history of hospitalization within the past 3 months, and were not institutionalized at the time of the survey, which may have mitigated this issue to some extent. Given the inherent limitations of observational studies, our sample likely included some individuals with dementia, in addition to the 30% who had already subjectively reported cognitive decline. Furthermore, individuals with unrecognized cognitive impairment may have also been included. Nevertheless, because participants were not institutionalized or in need of substantial care, it is reasonable to infer that their cognitive impairment was not severe enough to significantly interfere with daily life. Therefore, the extent of measurement bias is likely to have been relatively small. While previous studies have reported that individuals with dementia tend to rate their subjective health status poorly,^3^ it has also been noted that those with subjective cognitive decline do not necessarily exhibit such a tendency.^3^ Given these characteristics of our study population, we believe that measurement bias did not strongly affect the results, and, even if present, it likely resulted in only a slight overestimation.

With regard to the second concern – the need to examine comorbidities and medication use separately – we agree. These variables were combined in our study to reduce missing responses. Before this, we conducted separate analyses and found largely unchanged results. However, because detailed disease information was unavailable, future research should identify and incorporate conditions that may confound the relationship between weight loss and LTCI certification.

The third concern – handling malignancies as a critical confounder – is also well taken. Although we were unable to collect data on specific comorbid conditions, malignancies warrant careful consideration, as they have been reported to cause nearly 10% weight loss within a few months.^4^ While we acknowledge the limitation of lacking direct information on malignancies, our analytical approach may have accounted for this factor to some extent. Specifically, we employed Fine–Gray regression analysis, treating mortality as a competing risk when examining the association between weight loss and LTCI certification. Individuals with malignancies who experienced weight loss were likely to have a higher probability of mortality over the 9‐year follow‐up period.^4^ Thus, the Fine–Gray model may have adjusted for this phenomenon analytically. Moreover, under the Japanese healthcare system, individuals requiring care owing to terminal malignancies typically receive services under the medical insurance system rather than under the LTCI system. Consequently, those who experienced weight loss owing to malignancies may have transitioned directly to mortality without being captured as LTCI recipients. Such cases were likely accounted for through the Fine–Gray analysis. However, this remains a hypothesis that cannot be directly validated with our dataset, underscoring the need for further research.

Finally, concerning the background of weight loss and the obesity paradox, we acknowledge that these factors are critical for accurately interpreting our findings. The obesity paradox complicates the relationship between body mass index (BMI) and health outcomes, making it unclear whether BMI merely reflects adiposity or serves as an indicator of disease severity or progression. The lack of detailed disease information in our study represents a major limitation that may further obscure this paradox. Future studies should aim to collect comprehensive data on disease status, progression, and severity to elucidate what BMI truly represents in the context of aging and health. This approach will provide valuable public health insights.

We sincerely appreciate the valuable comments on our study. These insights have allowed for a more in‐depth discussion and will contribute to the advancement of future research. We are grateful for the thoughtful perspectives provided.

## Disclosure statement

The authors declare no conflicts of interest.

## Ethics approval statement

This study was approved by the ethics committees of the National Center for Geriatrics and Gerontology (no. 1012‐2) and Shiga University of Medical Science Research (no. R2017‐006).

## Data Availability

Data sharing is not applicable to this article as no new data were created or analyzed in this study.
